# Supplementary material for: Experiencing nature leads to healthier food choices
Source: Commun Psychol. 2024 Apr 2;2:24. doi: 10.1038/s44271-024-00072-x (PMC11332233; doi:10.1038/s44271-024-00072-x)
Supplement: Supplementary file 2 — Supplementary Information [file 44271_2024_72_MOESM2_ESM.pdf]

SUPPLEMENTARY NOTE 1: Past experimental research on exposure to nature and healthy eating

Supplementary Table 1. Summary of past research on exposure to nature and healthy eating

Article	Nature intervention	Key outcome variables	Summarized findings
1	Nature and urban groups: landscape photos.<br>Control: The Big Five Inventory questionnaire (no photos)	The amount of sugar chosen for a medium-sized bubble tea "reward beverage"<br><br>Delay discounting as a mediator	Participants (with the intention to lose weight) in the nature group chose less sugar in their bubble tea than did those in the urban or control groups.
2	Images of nature scenes or urban scenes	Delay discounting and food desirability scales for images of fruits and vegetables, and for images of energy-dense foods	No evidence of a difference in delay discounting nor food desirability scale ratings by condition
3	Nature group: family-based training program sessions teaching about the environment and food consumption behavior.<br>Control: reading government issued health-related dietary recommendations	Caregivers' scale assessments of their own and their children's level of connectedness to nature<br><br>Parental Feeding Style Questionnaire and Children's Eating Behavior Questionnaire	Participants in the nature group scored higher on connectedness to nature factors. Some improvements on food questionnaire factors, such as increased vegetable intake and (marginally) for fruit intake.
4	Slideshow images of green plants, green objects, greyscale plants, or greyscale objects, shown after exposed to a stressor	Snack cravings<br><br>Leeds Food Preference Questionnaire	Participants in the green plants condition exhibited the greatest decrease in snack cravings and the highest increase in implicit preference for vegetables.
5	Slideshow images of nature or urban environments in green or in white & black, shown after exposed to a stressor	Food wanting & liking on a 10-point scale<br><br>Snack food intake	No evidence of a difference between the nature and urban environments, nor in the green vs. white & black conditions, on food wanting, liking, nor intake
6	Posters with images of a grassland, trees and sky (nature condition), a runner (activity condition), a fair with carousels (fun condition), or no poster	Healthy vs. unhealthy vending machine snack choices	Participants in the nature condition made healthier snack choices when compared to those in the fun condition and control condition, but with no evidence of a difference when compared to the activity condition.
7	Restaurant field study with customary ambience as a baseline and a nature or fast-food ambience manipulated through changes in lighting, images, and sounds	Choice of vegetarian over meat-based lunch options	No evidence of a difference between the nature and fast-food ambience groups in their choice of vegetarian lunch options—and only a marginally significant difference between the nature and customary ambience groups.
8	Self-service buffet field experiment comparing nudge interventions (pre- vs. post-), where the "priming" nudge intervention consists of an eating environment filled with green plant imagery	Changes in meat-based chili, rice, vegetable, and total consumption	The "priming" condition reduced total energy intake through a decrease in meat consumption—but left vegetable and rice consumption unchanged.

SUPPLEMENTARY NOTE 2: Healthiness pilot test ratings for field study stimuli

The goal of this study was to rate the eight snack foods used as experimental stimuli in Study 1. Online respondents rated all eight foods on a 7-point scale of healthiness (from -3=*very unhealthy* to +3=*very healthy*) and naturalness (from -3=*very artificial* to +3=*very natural*). Using Amazon Mechanical Turk, 462 participants completed the survey, and 456 passed the attention check. Two participants were excluded because they gave the same answer to each question, leaving 454 respondents. The healthy food group (i.e., bananas, apples, dried fruits, and mixed nuts) was evaluated as higher in overall healthiness (M=6.04, SD=0.63) when compared to the unhealthy food group (M=2.22, SD=0.88; t=78.16, p<.001), which consisted of potato chips, brownies, strawberry cookies, and apricot cookies. Due to homogeneity in their participant ratings and nutritional profiles, the strawberry and apricot cookies (which were of the same brand and cookie type) were aggregated into one measure of simply "cookies". The naturalness ratings mirrored those of the healthiness ratings. The healthy foods group was evaluated as higher in overall naturalness (M=6.06, SD=0.66) when compared to the unhealthy foods group (M=2.33, SD=0.91; t=76.75, p<.001)."

SUPPLEMENTARY NOTE 3: Healthiness pilot test ratings for online study stimuli

This study assessed how participants perceive foods used in Studies 2, 3, and 5 in terms of healthiness. We recruited 100 online respondents on Prolific Academic. 17 participants failed the attention check and were therefore excluded from the analyses. We tested four drinks, four main courses, and four side dishes. Respondents assessed the foods and beverages on their own perceptions of healthiness on a 7-point Likert scale from *extremely unhealthy* to *extremely healthy*. The healthy drinks, mains, and sides were coconut water, water, cobb salad, salmon salad, Greek yogurt, and fruit salad, respectively. The unhealthy drinks, mains, and sides consisted of two types of sugar-sweetened sodas, a peanut butter and jelly sandwich, a hot dog, a chocolate candy bar, and potato chips, respectively. The healthy foods and beverages were evaluated as higher in overall healthiness (M=5.7, SD=0.7) when compared to the unhealthy selections (M=2.1, SD=0.6, t=41.4, p<.001).

SUPPLEMENTARY NOTE 4: Healthiness pilot test ratings for Study 4

We asked 100 online adult participants (U.S.-based, English-speaking) via Prolific to rate the three descriptions used in Study 4 (i.e., "a natural, healthy snack", "a diet, light snack", and "a tasty, indulgent snack") on healthiness (7-point scale from "not at all healthy" to "extremely healthy"). We obtained N=99 valid participants due to 1 attention check failure. The "natural, healthy snack" was rated most healthy (M=6.14, SD=1.00), followed by the "diet, light snack" (M=4.63, SD=1.36), and finally, the "tasty, indulgent snack" (M=2.32, SD=1.25). Paired t-tests show that the "natural, healthy snack" was perceived as significantly healthier than the "diet, light snack" (t(98)=10.38, p<.001, d=1.45), and significantly healthier than the "tasty, indulgent snack" (t(98)=25.31, p<.001, d=1.50). The "diet, light snack" was also perceived as significantly healthier than the "tasty, indulgent snack" (t(98)=14.38, p<.001, d=1.59).

SUPPLEMENTARY NOTE 5: Implicit association task (IAT)

We conducted a text-based implicit association task (IAT)[9] to explore whether the pictorial nature of the stimuli used in Studies 2-5 drives the effects of nature exposure, or whether there is a more general association between the concepts of nature and healthiness. The IAT assesses the strength of mental associations between two pairs of divergent concepts[10], such as nature vs. city and healthy vs. unhealthy attributes.

Iatgen[10], a survey software implementation tool, was used to conduct the IAT on Qualtrics. Two hundred U.S. residents (110 female) provided informed consent and completed the IAT online via Prolific Academic. The IAT employed the iatgen default settings with seven counterbalanced interactive blocks using text stimuli for targets and categories. "Nature" and "City" (used instead of *urban*, which is an adjective) composed the targets, while the categories consisted of "Healthy" and "Unhealthy". The IAT aims to determine the extent to which the target pairs, *nature* and *city* are mentally associated with the categories *healthy* and *unhealthy*.

The nature target comprised the following words: countryside, flower, forest, grass, hill, lake, leaf, meadow, mountain, ocean, river, and tree. The city target consisted of the following words: building, car, concrete, downtown, factory, highway, industry, metropolitan, noise, street, traffic, and urban. The healthy category featured the following terms: active, athletic, beneficial, fitness, life, nutritious, strong, thriving, vibrant, vitality, well-being, wellness—while the unhealthy category featured: dangerous, death, disease, feeble, harmful, illness, sick, stress, toxic, unwell, vulnerable, and weak. Default settings provided error feedback in the form of a red "x", forced users to correct errors, and provided a 250ms pause between trials.

The IAT data was analyzed using the iatgen web-based Shiny application, which provided key diagnostic variables[11]. Following past research, the IAT was *D*-scored, where positive values denote strong associations between nature and healthy[10,11]. Results confirmed the prediction and indicated a strong implicit association between nature and healthy, $M_{\text{D Score}} = 0.53$, $SD = 0.44$, $d = 1.22$, $p < .001$.

SUPPLEMENTARY NOTE 6: The Boundary Condition of Exposure to Photos of Nature and Urban Scenes in the Winter with Snow

In this pre-registered study (https://aspredicted.org/DTK_SW1), we recruited 1,380 U.K. residents via Prolific. Applying the preregistered exclusion criteria left a sample of 1,310 participants. Participants were randomly assigned to one of four conditions: a photo of a nature scene in the winter with snow, the same nature scene in the summer without snow, an urban scene in the winter with snow, and the same urban scene in the summer without snow. To test the robustness of the effects to the choice of stimuli, we used two photos for each of the four experimental conditions, counterbalanced across respondents. The remainder of the experiment follows consistently with the other studies, where participants were asked to describe the scene depicted in one sentence and to select a main course from a set of six dishes. Three dishes were healthy (salmon avocado bowl, Mediterranean chicken salad, and quinoa tomato bowl), and the other three were unhealthy (fish & chips, burger & fries, and macaroni & cheese). As preregistered, only participants who correctly recalled the season in the attention check were retained in the analyses.

In the summer condition, the proportion of healthy food choices was significantly higher after viewing the nature photo than after viewing the urban photo (M_{Nature}=56.9% vs.

M_{Urban}=47.2%; χ^2=5.82, p=.02). In contrast, the proportion of healthy choices did not differ when the photos were taken in the winter with snow (M_{Nature}=20.2% vs. M_{Urban}=20.8%; χ^2=.03, p=.87). Therefore, this study replicated the results of our prior studies with nature or urban scenes depicting mild/pleasant seasons but found that experiencing nature did not lead to healthier food choices—relative to an urban environment—when the photos were taken during a snowy winter.

SUPPLEMENTARY REFERENCES

1. Kao C-C, Wu W-H, Chiou W-B. Exposure to nature may induce lower discounting and lead to healthier dietary choices. *Journal of Environmental Psychology* 2019, **65:** 101333.

2. Clarke K, Higgs S, Holley CE, Jones A, Marty L, Hardman CA. A change of scenery: Does exposure to images of nature affect delay discounting and food desirability? *Frontiers in psychology* 2021**:** 5479.

3. Sobko T, Brown GT, Cheng WH. Does connectedness to nature improve the eating behaviours of pre-schoolers? Emerging evidence from the Play&Grow randomised controlled trial in Hong Kong. *Appetite* 2020, **154:** 104781.

4. Michels N, Debra G, Mattheeuws L, Hooyberg A. Indoor nature integration for stress recovery and healthy eating: A picture experiment with plants versus green color. *Environmental Research* 2022**:** 113643.

5. Michels N, De Witte F, Di Bisceglie E, Seynhaeve M, Vandebuerie T. Green nature effect on stress response and stress eating in the lab: Color versus environmental content. *Environmental Research* 2021, **193:** 110589.

6. Stöckli S, Stämpfli AE, Messner C, Brunner TA. An (un) healthy poster: When environmental cues affect consumers' food choices at vending machines. *Appetite* 2016, **96:** 368-374.

7. Vanhatalo S, Liedes H, Pennanen K. Nature ambience in a lunch restaurant has the potential to evoke positive emotions, reduce stress, and support healthy food choices and sustainable behavior: A field experiment among Finnish customers. *Foods* 2022, **11**(7)**:** 964.

8. Friis R, Skov LR, Olsen A, Appleton KM, Saulais L, Dinnella C*, et al.* Comparison of three nudge interventions (priming, default option, and perceived variety) to promote vegetable consumption in a self-service buffet setting. *PloS one* 2017, **12**(5)**:** e0176028.

9. Greenwald AG, McGhee DE, Schwartz JL. Measuring individual differences in implicit cognition: the implicit association test. *Journal of personality and social psychology* 1998, **74**(6)**:** 1464.

10. Carpenter TP, Pogacar R, Pullig C, Kouril M, Aguilar S, LaBouff J*, et al.* Survey-software implicit association tests: A methodological and empirical analysis. *Behavior research methods* 2019, **51**(5)**:** 2194-2208.

11. Greenwald AG, Nosek BA, Banaji MR. Understanding and using the implicit association test: I. An improved scoring algorithm. *Journal of personality and social psychology* 2003, **85**(2)**:** 197.
